# Function of Ltbp-4L and fibulin-4 in survival and elastogenesis in mice

**DOI:** 10.1242/dmm.026005

**Published:** 2016-11-01

**Authors:** Insa Bultmann-Mellin, Jeroen Essers, Paula M. van Heijingen, Harald von Melchner, Gerhard Sengle, Anja Sterner-Kock

**Affiliations:** 1 Center for Experimental Medicine, Medical Faculty, University of Cologne, 50931 Cologne, Germany; 2 Department of Molecular Genetics, Cancer Genomics Centre, Erasmus MC, 3015 CN Rotterdam, The Netherlands; 3 Department of Radiation Oncology, Erasmus MC, 3015 CN Rotterdam, The Netherlands; 4 Department of Vascular Surgery, Erasmus MC, 3015 CN Rotterdam, The Netherlands; 5 Department of Molecular Hematology, University of Frankfurt Medical School, 60590 Frankfurt am Main, Germany; 6 Center for Biochemistry, Medical Faculty, University of Cologne, 50931 Cologne, Germany; 7 Center for Molecular Medicine Cologne (CMMC), University of Cologne, 50931 Cologne, Germany

**Keywords:** Latent-transforming growth factor beta-binding protein 4 (Ltbp-4), Fibulin-4, Elastic fibers, Defective alveolar septation, Aortic tortuosity

## Abstract

LTBP-4L and LTBP-4S are two isoforms of the extracellular matrix protein latent-transforming growth factor beta-binding protein 4 (LTBP-4). The mutational inactivation of both isoforms causes autosomal recessive cutis laxa type 1C (ARCL1C) in humans and an ARCL1C-like phenotype in *Ltbp4*^−/−^ mice, both characterized by high postnatal mortality and severely affected elastogenesis. However, genetic data in mice suggest isoform-specific functions for Ltbp-4 because *Ltbp4S*^−/−^ mice, solely expressing Ltbp-4L, survive to adulthood. This clearly suggests a requirement of Ltbp-4L for postnatal survival. A major difference between *Ltbp4S*^−/−^ and *Ltbp4*^−/−^ mice is the matrix incorporation of fibulin-4 (a key factor for elastogenesis; encoded by the *Efemp2* gene), which is normal in *Ltbp4S*^−/−^ mice, whereas it is defective in *Ltbp4*^−/−^ mice, suggesting that the presence of Ltbp-4L might be required for this process. To investigate the existence of a functional interaction between Ltbp-4L and fibulin-4, we studied the consequences of fibulin-4 deficiency in mice only expressing Ltbp-4L*.* Resulting *Ltbp4S*^−/−^;*Fibulin-4*^R/R^ mice showed a dramatically reduced lifespan compared to *Ltbp4S*^−/−^ or *Fibulin-4*^R/R^ mice, which survive to adulthood. This dramatic reduction in survival of *Ltbp4S*^−/−^;*Fibulin-4*^R/R^ mice correlates with severely impaired elastogenesis resulting in defective alveolar septation and distal airspace enlargement in lung, and increased aortic wall thickness with severely fragmented elastic lamellae. Additionally, *Ltbp4S*^−/−^;*Fibulin-4*^R/R^ mice suffer from aortic aneurysm formation combined with aortic tortuosity, in contrast to *Ltbp4S*^−/−^ or *Fibulin-4*^R/R^ mice. Together, in accordance with our previous biochemical findings of a physical interaction between Ltbp-4L and fibulin-4, these novel *in vivo* data clearly establish a functional link between Ltbp-4L and fibulin-4 as a crucial molecular requirement for survival and elastogenesis in mice.

## Abstract (precis)

**Summary:** The interaction of the long form of latent-transforming growth factor beta-binding protein 4 and fibulin-4 is essential for survival as well as for the formation of elastic fibers.

## INTRODUCTION

Latent-transforming growth factor beta-binding protein 4 (LTBP-4) is expressed, secreted and deposited within the extracellular matrix (ECM) as a short (LTBP-4S) and a long (LTBP-4L) isoform (Robertson et al., 2015). In humans and mice, both isoforms show distinct tissue expression patterns in various organs (Bultmann-Mellin et al., 2015; Kantola et al., 2010), which has been proposed to result from the activation of two known *LTBP-4* promoters under the control of independent transcription factors (Bultmann et al., 2013; Kantola et al., 2010).

In humans, mutations in the *LTBP4* gene cause functional modifications of both LTBP-4 isoforms, leading to autosomal recessive cutis laxa type 1C (ARCL1C; initially called Urban-Rifkin-Davis syndrome). ARCL1C is a rare congenital connective tissue disorder characterized by high mortality in the first months of life due to impaired elastic fiber architecture in several visceral organs, including massive emphysemas in the lung leading to early death by respiratory failure (Callewaert et al., 2013; Urban et al., 2009).

We previously generated *Ltbp4*-null (*Ltbp4*^−/−^) mice in which the expression of both Ltbp-4 isoforms (Ltbp-4S, NM_001113549.1; Ltbp-4L, NM_175641.2) is ablated (Bultmann-Mellin et al., 2015). In contrast to mice only expressing Ltbp-4L (*Ltbp4S*^−/−^) (Dabovic et al., 2009; Sterner-Kock et al., 2002), *Ltbp4*^−/−^ mice nearly replicate the features of ARCL1C syndrome, including high postnatal mortality, impaired pulmonary lobular architecture with emphysematous and atelectatic areas, and impaired elastic fiber formation, suggesting that the presence of LTBP-4L is required for elastic fiber formation, intact pulmonary architecture and survival (Bultmann-Mellin et al., 2015).

*In vitro* studies, using human fibroblasts, demonstrated that LTBP-4S potentiates the formation of elastic fibers through a functional interaction with fibulin-5 (FBLN5), a tropoelastin-binding protein necessary for elastogenesis (Noda et al., 2013). However, recent data show that *Ltbp4S*^−/−^;*Fbln5*^−/−^ mice, which express Ltbp-4L and lack fibulin-5 expression, have fibrillar intact elastic fibers. This finding leads to the conclusion that an alternative elastogenesis pathway might exist, most likely involving fibulin-4 [encoded by the *Efemp2* (epidermal growth factor-containing fibulin-like extracellular matrix protein 2) gene] (Dabovic et al., 2015). Fibulin-4 is required for proper elastic fiber assembly and is highly expressed in the medial layers of blood vessel walls, including the aortic media (Hucthagowder et al., 2006; McLaughlin et al., 2006). Mice lacking fibulin-4 (*Fibulin-4*^−/−^) die perinatally from aortic rupture (McLaughlin et al., 2006), but mice with a systemic reduction of fibulin-4 expression (*Fibulin-4*^R/R^) survive until adulthood, indicating that a minimal amount of fibulin-4 is required for survival. Similar to individuals with mutations in the gene coding for fibulin-4, *Fibulin-4*^R/R^ mice display elastic fiber fragmentation and develop aortic aneurysms (Hanada et al., 2007; Hucthagowder et al., 2006).

Our previous studies identify fibulin-4 as a newly described interaction partner for both Ltbp-4 isoforms, with isoform-specific differences regarding their molecular specificity. Both Ltbp-4L and Ltbp-4S bind to fibulin-4 via their N-termini. However, compared with Ltbp-4S, we measured a higher binding propensity for Ltbp-4L to fibulin-4. The observed differences in binding propensity are probably important, because incorporation of fibulin-4 into the matrix remains unaltered in the presence of Ltbp-4L in *Ltbp4S*^−/−^ mice, whereas it is defective in *Ltbp4*^−/−^ mice, where both Ltbp-4 isoforms are lacking (Bultmann-Mellin et al., 2015). *Ltbp4*^−/−^ mice die between postnatal day (P)8 and P14 (Bultmann-Mellin et al., 2015), whereas *Ltbp4S*^−/−^ mice survive to adulthood (Sterner-Kock et al., 2002), indicating that Ltbp-4L expression and possibly its interaction with fibulin-4 is crucial for survival of *Ltbp4S*^−/−^ mice.

To test the existence of a functional interaction between Ltbp-4L and fibulin-4, we studied the consequences of fibulin-4 deficiency in mice only expressing Ltbp-4L. For this purpose, we crossed *Ltbp4S*^+/−^ mice (Sterner-Kock et al., 2002) with *Fibulin-4*^+/R^ mice (Hanada et al., 2007; Ramnath et al., 2014) to generate and analyze *Ltbp4S*^−/−^;*Fibulin-4*^R/R^ mice, which only express the Ltbp-4L isoform and show reduced fibulin-4 expression.

## RESULTS

### Clinical and pathological findings of *Ltbp4S*^−/−^;*Fibulin-4*^R/R^ mice with reduced interaction of Ltbp-4L and fibulin-4

**Fig. 1.**
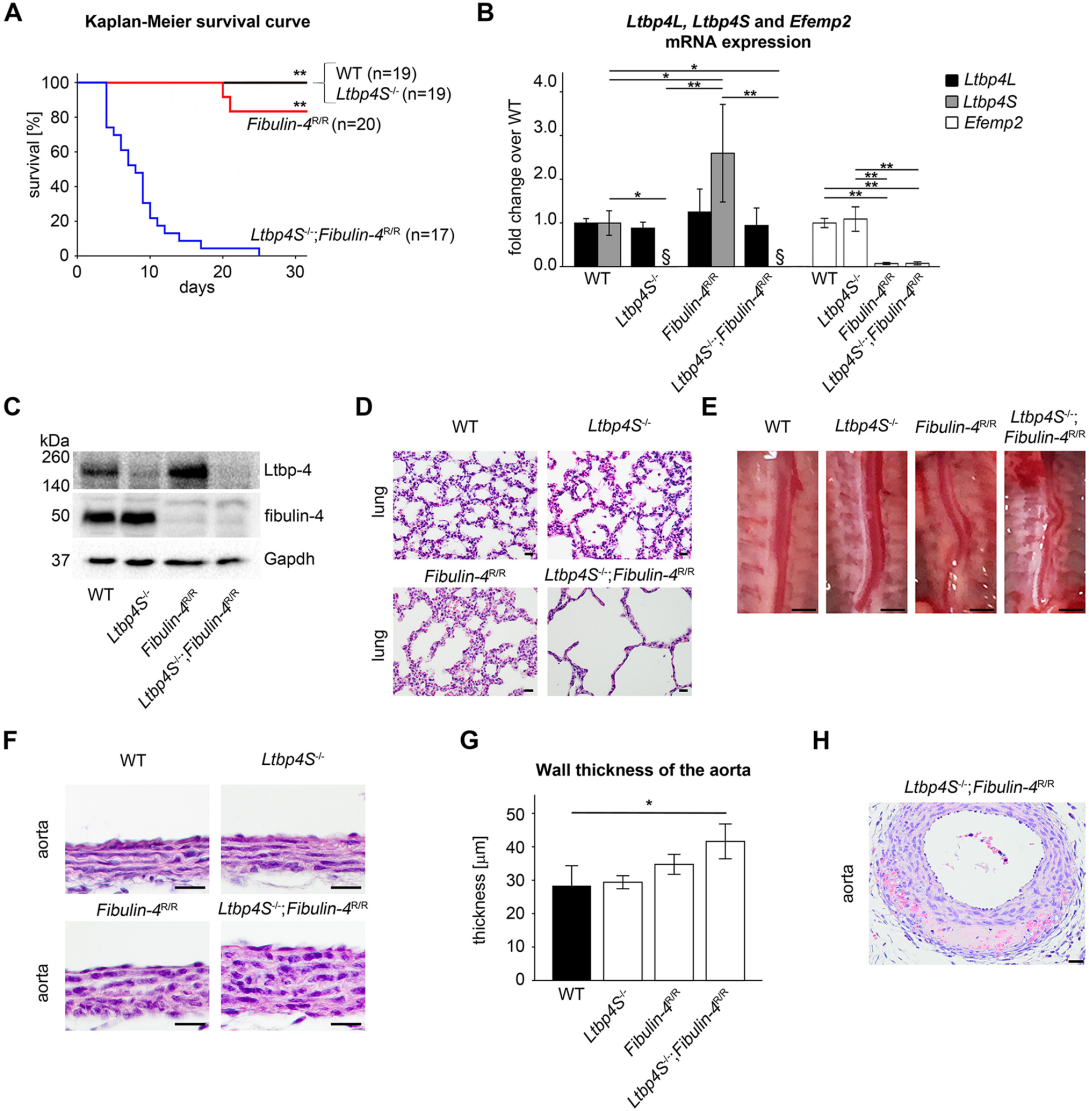
**Clinical and pathological findings of *Ltbp4S*^−/−^;*Fibulin-4*^R/R^ mice with reduced interaction of Ltbp-4L and fibulin-4.** (A) Kaplan–Meier survival curve revealed significantly higher neonatal mortality in *Ltbp4S*^−/−^;*Fibulin-4*^R/R^ mice (*n*=17) compared to WT (*n*=19), *Ltbp4S*^−/−^ (*n*=19) and *Fibulin-4*^R/R^ (*n*=20) mice, which showed no differences in survival. Differences between groups were analyzed by log-rank test (**P<0.01 vs *Ltbp4S*^−/−^;*Fibulin-4*^R/R^). (B) Quantitative PCRs and (C) representative immunoblots showed *Ltbp-4* and *Efemp2* mRNA and Ltbp-4 and fibulin-4 protein expression of WT (*n*=3), *Ltbp4S*^−/−^ (*n*=3), *Fibulin-4*^R/R^ (*n*=4) and *Ltbp4S*^−/−^;*Fibulin-4*^R/R^ (*n*=4) lungs. Differences between groups were analyzed by two-way ANOVA, followed by Bonferroni correction (*P<0.05 and **P<0.01; ^§^below detection limit). (D) In *Ltbp4S*^−/−^ and *Fibulin-4*^R/R^ mice, the pulmonary parenchyma had enlarged alveolar spaces with reduced numbers of alveoli compared to WT mice. Lungs of *Ltbp4S*^−/−^;*Fibulin-4*^R/R^ mice showed a higher degree of severely enlarged alveolar spaces with multifocal areas of atelectasis and a lack of alveolar and lobular architecture compared to all other genotypes. Scale bars: 20 µm. (E) *Ltbp4S*^−/−^, *Fibulin-4*^R/R^ and *Ltbp4S*^−/−^;*Fibulin-4*^R/R^ mice displayed tortuous aortas. Scale bars: 10 mm. (F,G) Abdominal aortas showed marked thickening of the aortic wall in *Ltbp4S*^−/−^;*Fibulin-4*^R/R^ mice compared to the other genotypes. Differences between groups were analyzed by two-way ANOVA, followed by Bonferroni correction (*n*=3; *P<0.05; scale bars: 20 µm). (H) Abdominal aortas of *Ltbp4S*^−/−^;*Fibulin-4*^R/R^ mice showed intramural hemorrhages and destruction of the aortic wall with necrotic cellular debris (scale bar: 20 µm). Data are presented as means±s.d.; *n* indicates the number of analyzed tissue of individual mice or analyzed mice.

To investigate whether a functional interdependence between Ltbp-4L and fibulin-4 is crucial for the survival of mice, *Ltbp4S*^+/−^ mice (Sterner-Kock et al., 2002) were crossed with *Fibulin-4*^+/R^ mice (Hanada et al., 2007; Ramnath et al., 2014). Resulting wild-type (WT), *Ltbp4S*^−/−^, *Fibulin-4*^R/R^ and *Ltbp4S*^−/−^;*Fibulin-4*^R/R^ mice were analyzed. These mice were born according to the Mendelian inheritance pattern (Table 1). *Ltbp4S*^−/−^;*Fibulin-4*^R/R^ mice died between P4 and P8 (Fig. 1A and Table 1). This fact represented a dramatically reduced lifespan compared to WT, *Ltbp4S*^−/−^ and *Fibulin-4*^R/R^ mice. *Ltbp4S*^−/−^ mice survive to adulthood (Sterner-Kock et al., 2002) and *Fibulin-4*^R/R^ mice survive up to 3-4 months, when avoiding any stress (Ramnath et al., 2014). *Ltbp4S*^−/−^;*Fibulin-4*^R/R^ mice reacted extremely sensitively to stress. About 50% of *Ltbp4S*^−/−^;*Fibulin-4*^R/R^ mice died suddenly during handling procedures starting at the age of 3-4 days postnatally (these mice did not contribute to the survival curve). Necropsy revealed extensive hemorrhages in the thoracic region most likely due to a disrupted aorta. All analyses were performed at P4. At this age there was no difference in body weight between the genotypes (Table 1).

**Table 1.**
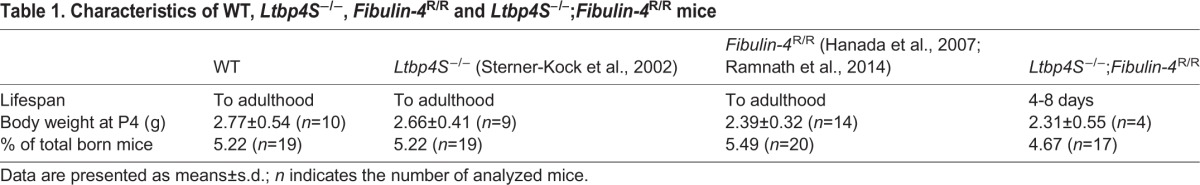
**Characteristics of WT, *Ltbp4S*^−/−^, *Fibulin-4*^R/R^ and *Ltbp4S^−/−^*;*Fibulin-4*^R/R^ mice**

To verify the genetic background of WT, *Ltbp4S*^−/−^, *Fibulin-4*^R/R^ and *Ltbp4S*^−/−^;*Fibulin-4*^R/R^ mice, we analyzed mRNA and protein expression of Ltbp-4 and fibulin-4. Owing to the lack of Ltbp-4 isoform-specific antibodies, Ltbp-4 protein expression and deposition was investigated with an antibody recognizing both isoforms. Lungs of WT, *Ltbp4S*^−/−^, *Fibulin-4*^R/R^ and *Ltbp4S*^−/−^;*Fibulin-4*^R/R^ mice showed no differences in *Ltbp4L* mRNA expression (Fig. 1B). *Ltbp4S* mRNA expression was below detection limit in *Ltbp4S*^−/−^ and *Ltbp4S*^−/−^;*Fibulin-4*^R/R^ lungs. *Ltbp4S* mRNA and Ltbp-4 protein expression was upregulated in *Fibulin-4*^R/R^ lungs compared to all other genotypes (Fig. 1B,C). *Ltbp4S*^−/−^ and *Ltbp4S*^−/−^;*Fibulin-4*^R/R^ lungs expressed about 10% Ltbp-4 protein compared to WT lungs. This residual expression reflects the amount of Ltbp-4L protein in these lungs (Fig. 1C). Lungs of *Ltbp4S*^−/−^ mice showed no difference in *Efemp2* mRNA expression compared to WT lungs. *Fibulin-4*^R/R^ and *Ltbp4S*^−/−^;*Fibulin-4*^R/R^ lungs showed about 10% *Efemp2* mRNA expression and about 10% fibulin-4 protein expression compared to WT lungs (Fig. 1B,C).

In lungs from all analyzed genotypes, Ltbp-4 and fibulin-4 immunoreactivity was found in the bronchial and bronchiolar walls, in the pulmonary parenchyma and vascular walls (Fig. S1). Lungs from *Ltbp4S*^−/−^, *Fibulin-4*^R/R^ and *Ltbp4S*^−/−^;*Fibulin-4*^R/R^ mice showed enlarged alveolar spaces with reduced numbers of alveoli compared to WT lungs (Fig. 1D). However, lungs of *Ltbp4S*^−/−^;*Fibulin-4*^R/R^ mice showed a higher degree of severely enlarged alveolar spaces with multifocal areas of atelectasis and a lack of alveolar and lobular architecture compared to all other genotypes (Fig. 1D).

In aortas of all genotypes, Ltbp-4 immunoreactivity was present throughout the entire aortic wall, especially in the vicinity of the aortic elastic lamellae. Fibulin-4 immunoreactivity was also found throughout the entire aortic wall, but was predominantly evident in the vicinity of the adventitia in aortas of all genotypes, and was strongly reduced in *Fibulin-4*^R/R^ and *Ltbp4S*^−/−^;*Fibulin-4*^R/R^ mice (Fig. S1). *Ltbp4S*^−/−^, *Fibulin-4*^R/R^ and *Ltbp4S*^−/−^;*Fibulin-4*^R/R^ mice displayed malformed and tortuous aortas with dilatation of the lumen of the abdominal aortas (Fig. 1E). Aortic walls of *Ltbp4S*^−/−^;*Fibulin-4*^R/R^ mice were significantly thicker compared to those of WT mice. Aortic walls of *Ltbp4S*^−/−^;*Fibulin-4*^R/R^ mice showed a markedly increased thickness compared to *Ltbp4S*^−/−^ and *Fibulin-4*^R/R^ mice (Fig. 1F,G) and revealed focally extensive intramural hemorrhages and destruction of the aortic wall with necrotic cellular debris, which can be associated with lesions of aortic aneurysms (Fig. 1H). These pathological changes were not observed in the aortas of WT, *Ltbp4S*^−/−^ or *Fibulin-4*^R/R^ mice at P4 (Fig. 1F).

### Impaired elastogenesis in *Ltbp4S*^−/−^;*Fibulin-4*^R/R^ mice with reduced interaction of Ltbp-4L and fibulin-4

**Fig. 2.**
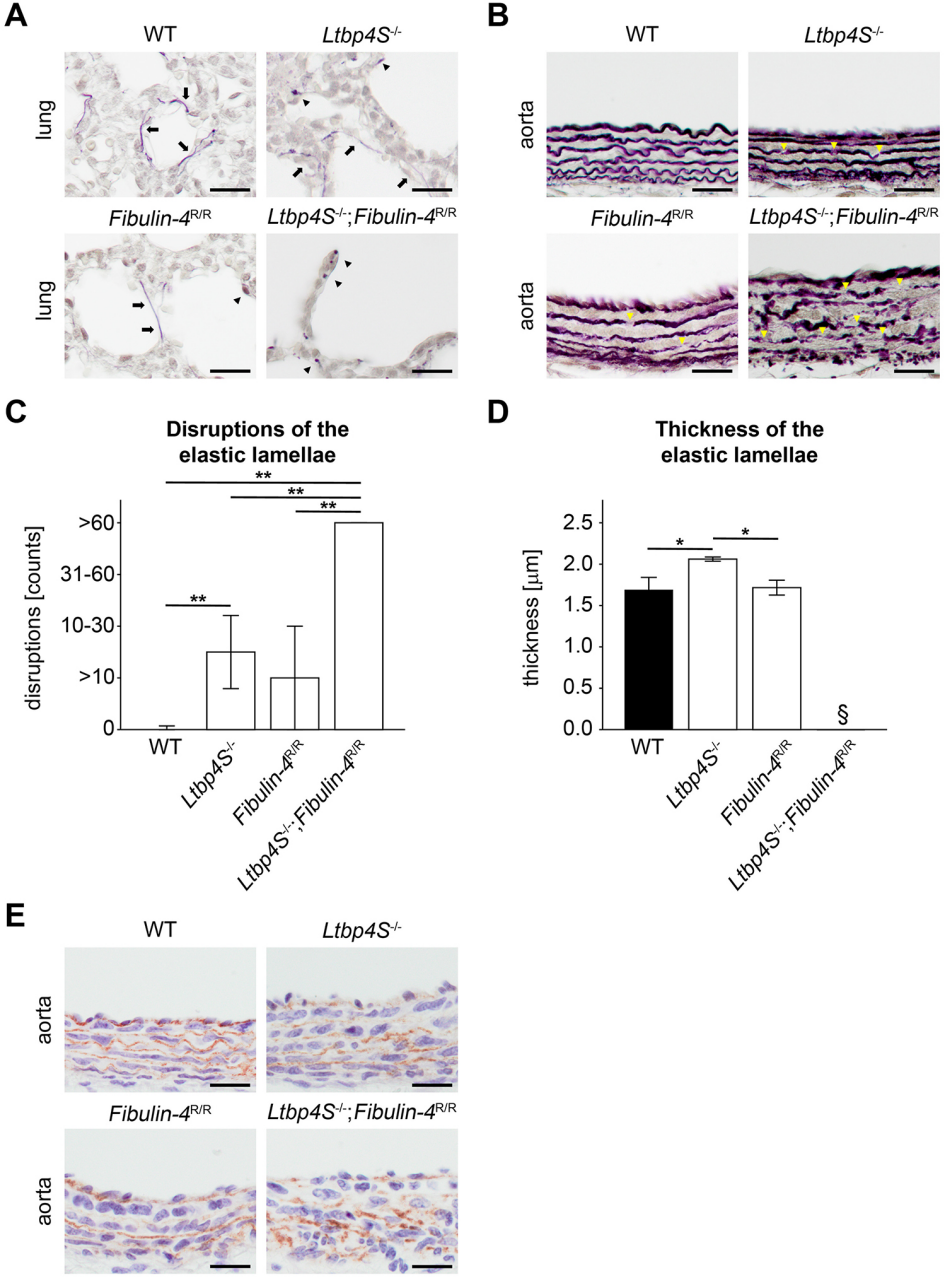
**Impaired elastogenesis in *Ltbp4S*^−/−^;*Fibulin-4*^R/R^ mice with reduced interaction of Ltbp-4L and fibulin-4.** (A) Representative histochemical elastica stainings of lungs showed intact elastic fibers (arrows) in WT mice. In *Ltbp4S*^−/−^ and *Fibulin-4*^R/R^ mice, elastic fibers were composed of both intact fibers (arrows) and scattered patches of elastin aggregates (arrowheads). Elastic fibers were severely fragmented (arrowheads) in *Ltbp4S*^−/−^;*Fibulin-4*^R/R^ mice (scale bars: 20 µm). (B) Representative histochemical elastica stainings of abdominal aortas showed occasional disruptions (yellow arrowheads) in *Fibulin-4*^R/R^ mice and moderate elastic fiber fragmentations (yellow arrowheads) in *Ltbp4S*^−/−^ mice. In *Ltbp4S*^−/−^;*Fibulin-4*^R/R^ mice, elastic fibers were severely fragmented (yellow arrowheads) (scale bars: 20 µm). (C) Quantitative analysis of disruptions of the medial elastic lamellae showed significantly higher numbers of disruptions in *Ltbp4S*^−/−^ abdominal aortas compared to WT abdominal aortas and significantly higher numbers of disruptions in *Ltbp4S*^−/−^;*Fibulin-4*^R/R^ aortas compared to all other genotypes (*n*=3; **P<0.01). (D) Quantitative analysis of the thickness of the medial elastic lamellae showed significantly thicker elastic lamellae in *Ltbp4S*^−/−^ abdominal aortas compared to WT and *Fibulin-4*^R/R^ abdominal aortas. The thickness of the medial elastic lamellae could not be determined in *Ltbp4S*^−/−^;*Fibulin-4*^R/R^ abdominal aortas (*n*=3; *P<0.05; ^§^not determinable). (E) Representative images showed fibulin-5 immunoreactivity and disruption of the fibrillar structure of fibulin-5 fibers in abdominal aortas of *Ltbp4S*^−/−^ and *Ltbp4S*^−/−^;*Fibulin-4*^R/R^ mice compared to WT and *Fibulin-4*^R/R^ abdominal aortas (scale bars: 20 µm). Data are presented as means±s.d.; *n* indicates the number of analyzed tissue of individual mice. Differences between groups were analyzed by two-way ANOVA, followed by Bonferroni correction.

WT mice showed intact elastic fibers in lungs (Fig. 2A; black arrows). The elastic fibers in *Ltbp4S*^−/−^ and *Fibulin-4*^R/R^ mice were composed of both intact elastic fibers (Fig. 2A; black arrows) and scattered patches of elastin aggregates (Fig. 2A; black arrowheads). The elastic fibers of *Ltbp4S*^−/−^;*Fibulin-4*^R/R^ mice were severely fragmented (Fig. 2A; black arrowheads). The protein expression of tropoelastin showed no differences in lungs of all genotypes (Fig. S2). The elastic lamellae in aortas of *Fibulin-4*^R/R^ mice showed occasional disruptions compared to WT mice (Fig. 2B,C; yellow arrowheads; *P*=n.s.). There were moderate fragmentations with intact and disrupted elastic lamellae in the aortas of *Ltbp4S*^−/−^ mice (Fig. 2B,C; yellow arrowheads; *P*<0.01 vs WT mice). The elastic lamellae of WT aortas appeared more wavy than elastic lamellae of *Ltbp4S*^−/−^ and *Fibulin-4*^R/R^ aortas (Fig. 2B). *Ltbp4S*^−/−^;*Fibulin-4*^R/R^ mice showed no intact elastic lamellae in the aortas (Fig. 2B,C; yellow arrowheads; *P*<0.01 vs all other genotypes). The thickness of the medial elastic lamellae was thicker in *Ltbp4S*^−/−^ mice compared to WT and *Fibulin-4*^R/R^ mice (Fig. 2D; *P*<0.05). In aortas of *Ltbp4S*^−/−^;*Fibulin-4*^R/R^ mice, the thickness of the medial elastic lamellae could not be determined because no intact elastic fiber was present (Fig. 2B).

It has been demonstrated that Ltbp-4 isoforms functionally interact with fibulin-5 (Bultmann-Mellin et al., 2015; Noda et al., 2013). The fibrillar structure of fibulin-5 appeared normal in aortas of WT and *Fibulin-4*^R/R^ mice (Fig. 2E). In *Ltbp4S*^−/−^ and *Ltbp4S*^−/−^;*Fibulin-4*^R/R^ mice, the normal fibrillar structure was replaced by scattered amorphous patches of fibulin-5 (Fig. 2E). The protein expression of fibulin-5 showed no differences between lungs of all genotypes (Fig. S2).

## DISCUSSION

In this study, we investigated whether a functional interdependence between Ltbp-4L and fibulin-4 influences survival and phenotype of mice. Therefore, *Ltbp4S*^−/−^;*Fibulin-4*^R/R^ mice were generated, which only express the Ltbp-4L isoform and show reduced fibulin-4 expression.

In contrast to *Ltbp4S*^−/−^ and *Fibulin-4*^R/R^ mice, which survive to adulthood (Hanada et al., 2007; Ramnath et al., 2014; Sterner-Kock et al., 2002), *Ltbp4S*^−/−^;*Fibulin-4*^R/R^ mice died in the early postnatal period (P4-P8). The observed lung and aortic phenotype was more severe in the *Ltbp4S*^−/−^;*Fibulin-4*^R/R^ mice compared to *Ltbp4*^−/−^ mice at P4 (Fig. S3). This was most likely causative for the higher postnatal mortality of *Ltbp4S*^−/−^;*Fibulin-4*^R/R^ mice compared to *Ltbp4*^−/−^ mice (Bultmann-Mellin et al., 2015).

Both *Ltbp4S*^−/−^ and *Fibulin-4*^R/R^ mice show only mild emphysema in the early postnatal period and develop severe emphysema later in life (Bultmann-Mellin et al., 2015; Ramnath et al., 2014; Sterner-Kock et al., 2002). *Ltbp4S*^−/−^;*Fibulin-4*^R/R^ lungs showed already severe emphysema at P4, indicating that the mutual presence of both proteins (Ltbp-4L and fibulin-4) is essential for normal lung development. It has been described that fibrillin-1-deficient (*Fbn1*^mgΔ/mgΔ^) mice display impaired distal alveolar septation in the early postnatal period and develop emphysema at an older age (Neptune et al., 2003). In lungs of *Fbn1*^mgΔ/mgΔ^ mice, deposition and localization of elastin is normal. Additionally, increased activation of TGFβ signaling is found to be causative for the lung phenotype because it could be attenuated by perinatal application of a TGFβ-neutralizing antibody (Neptune et al., 2003). However, we found normal matrix deposition of fibrillin-1 (Fig. S4) with no alterations in expression levels of TGFβ downstream targets (*Ctgf* and *Pai1*; Fig. S5) in lungs of *Ltbp4S*^−/−^, *Fibulin-4*^R/R^ and *Ltbp4S*^−/−^;*Fibulin-4*^R/R^ mice at P4. These data suggest that altered fibrillin-1 fiber structure or matrix deposition did not contribute to the development of emphysema in *Ltbp4S*^−/−^, *Fibulin-4*^R/R^ or *Ltbp4S*^−/−^;*Fibulin-4*^R/R^ mice.

In humans or mouse models with arterial tortuosity syndrome (Cheng et al., 2009; Coucke et al., 2006), Loeys-Dietz syndrome (Gallo et al., 2014; Loeys et al., 2006), Marfan syndrome (Morris et al., 2011), with mutations in genes of the TGFβ signaling pathway [*SMAD3* (van de Laar et al., 2011, 2012), *TGFB2* (Lindsay et al., 2012), *PRKG1* (Guo et al., 2013)] or with mutations in genes directly involved in elastogenesis [*EFEMP2* (Hebson et al., 2014), *Fbln5* (Nakamura et al., 2002; Yanagisawa et al., 2002), *Ltbp4* (Bultmann-Mellin et al., 2015), *Eln* (Wagenseil et al., 2009)], aortic or arterial tortuosity is a commonly described feature. Increased aortic tortuosity is associated with a poorer prognosis in aortic diseases (Franken et al., 2015; Hatakeyama et al., 2001; Morris et al., 2011; Shirali et al., 2013), but the detailed mechanisms leading to tortuosity are still unknown (Morris, 2015). However, elastic fibers are altered in most cases of aortic tortuosity (Morris, 2015). Our finding of a functional interaction between Ltbp-4L and fibulin-4 might allow further insight into the pathogenesis for this condition to be gained.

TGFβ signaling is upregulated in aortas of adult *Fibulin-4*^R/R^ mice and its postnatal inhibition with angiotensin (Ang)II type 1 (AT1) receptor antagonist losartan improves lifespan of *Fibulin-4*^R/R^ mice, but does not affect aortic vessel wall structure. Prenatal treatment with losartan prevents elastic fiber fragmentation in the aortic media of *Fibulin-4*^R/R^ mice, indicating that altered TGFβ signaling is associated with disturbed elastic fiber structure (Moltzer et al., 2011). However, mRNA expression of TGFβ downstream targets (*Ctgf* and *Pai1*) showed no differences in aortas from WT, *Ltbp4S*^−/−^, *Fibulin-4*^R/R^ and *Ltbp4S*^−/−^;*Fibulin-4*^R/R^ mice at P4 (Fig. S5), but this might be different in other age groups.

Analysis of the aortae of all genotypes revealed that disrupted aortic elastic lamellae appeared to be thicker than continuous lamellae. *Fibulin-4*^R/R^ mice showed only occasional disruptions and no thickening of the elastic lamellae compared to WT mice. In contrast, the aortic elastic lamellae were significantly disrupted and significantly thicker in *Ltbp4S*^−/−^ mice compared to WT. A possible explanation for the described thickening of the elastic lamellae might be the presence of non-linearized tropoelastin, which spontaneously aggregates to form large globular structures *in vitro* (Tu and Weiss, 2010).

*Ltbp4S*^−/−^;*Fibulin-4*^R/R^ mice displayed aortic wall thickening compared to WT mice. It is very likely that deposition of amorphous, proteinaceous material within the aortic wall was the cause for the increased aortic thickness, a feature that has already been described in *Ltbp4*^−/−^ (Bultmann-Mellin et al., 2015) and *Fibulin-4*^R/R^ (Hanada et al., 2007; Moltzer et al., 2011) mice.

*Ltbp4S*^−/−^;*Fibulin-4*^R/R^ mice showed an increased mortality related to handling at P3-P4. These mice displayed intramural aortic hemorrhages and destruction of the aortic wall with intramural necrotic cellular debris (Fig. 1H). Aortic aneurysm formation and dissection is also described for adult *Fibulin-4*^R/R^ mice (Hanada et al., 2007). However, studies in humans also imply a role for LTBP-4 in this condition. Data from four geographically distinct case control studies showed that sizes and growth rate of abdominal aortic aneurysm significantly correlate with the presence of a particular *LTBP4* single-nucleotide polymorphism (*LTBP4* 21011A>T genotype). These data indicate a possible contribution of LTBP-4 to abdominal aortic aneurysm progression (Thompson et al., 2010). Therefore, ablation of Ltbp-4L in a *Fibulin-4^R/R^* background might further promote aneurysm formation and dissection in *Ltbp4S*^−/−^;*Fibulin-4*^R/R^ mice.

Fibulin-4 expression is not only essential for elastogenesis but also for intact collagen fiber assembly and homeostasis. Mice with smooth-muscle-specific loss of fibulin-4 expression show altered fibrillar collagen localization with larger, poorly organized fibrils in aortic walls (Papke et al., 2015) and fibulin-4 knockout mice show upregulation of the neutrophil collagenase matrix metalloprotease-8 in aortic walls (Kim et al., 2015). It is possible that impaired collagen assembly or homeostasis might contribute to the aortic phenotype in *Ltbp4S*^−/−^;*Fibulin-4*^R/R^ mice, which is under current investigation.

Mutations in the human *EFEMP2* gene lead to ARCL1B, whereas mutations in the human *LTBP4* gene are causative for ARCL1C (Callewaert and Urban, 2016; Loeys et al., 2015). There are several clinical similarities between ARCL1B and ARCL1C patients, such as cutis laxa, and craniofacial and pulmonary phenotypes, indicating a functional relationship between both genes. However, abnormal gastrointestinal and urinary development is only present in individuals with ARCL1C, in which pulmonary abnormalities seem to develop earlier and are more pronounced than in those with ARCL1B. Individuals with ARCL1B, on the other hand, display cardiovascular aberrations, involving arterial tortuosity, aneurysms and stenosis, which are not seen in ARCL1C patients (Callewaert and Urban, 2016; Loeys et al., 2015). *Fibulin-4*^R/R^ mice closely model ARCL1B, including severe cardiovascular abnormalities (Hanada et al., 2007; Moltzer et al., 2011). Contrary to *Ltbp4*^−/−^ mice, which resemble the features of ARCL1C, *Ltbp4S*^−/−^ mice develop a milder form of ARCL1C with a later onset of symptoms and prolonged survival (Bultmann-Mellin et al., 2015). The generated *Ltbp4S*^−/−^;*Fibulin-4*^R/R^ mice displayed very pronounced symptoms of both ARCL1B and ARCL1C, indicating the interdependence between Ltbp-4L and fibulin-4.

**Fig. 3.**
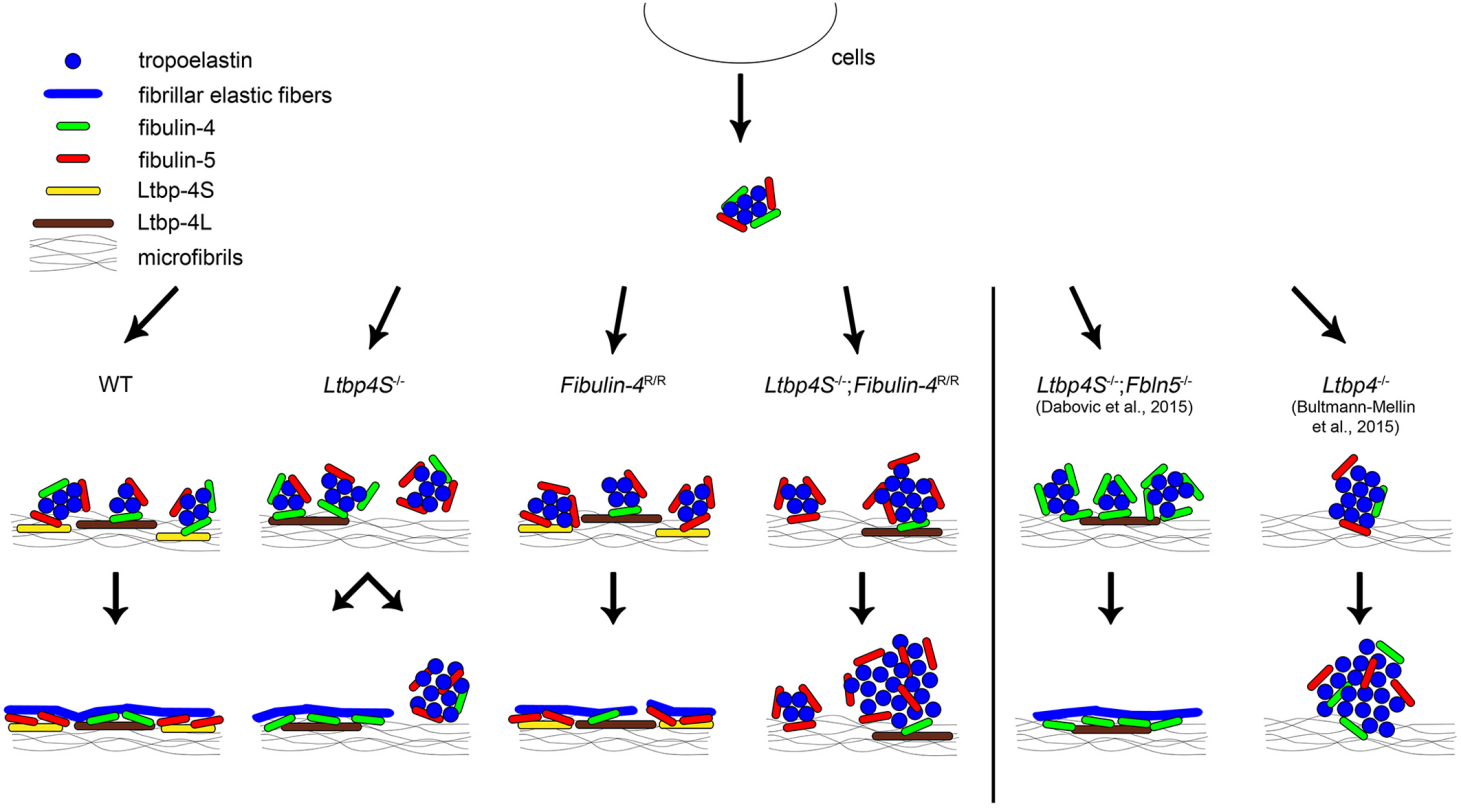
**Proposed model for the role of Ltbp-4L, Ltbp-4S, fibulin-4 and fibulin-5 in elastogenesis.** Normal elastogenesis in WT mice: proper elastic fiber assembly requires microaggregation of tropoelastin linked to fibulin-4 and fibulin-5 followed by binding of this complex to Ltbp-4L and Ltbp-4S and its linear deposition onto fibrillin microfibrils. Ltbp-4L might favor the interaction with fibulin-4 and Ltbp-4S with fibulin-5. The subsequent coalescence of tropoelastin results in fibrillar elastic fibers. *Ltbp4S*^−/−^ mice: fibulin-4, as part of the tropoelastin complex, binds to Ltbp-4L, resulting in scattered fibrillar elastic fibers. Fibulin-5 cannot bind to Ltbp-4S, which leads to amorphous, non-fibrillar elastin aggregates. *Fibulin-4*^R/R^ mice: if fibulin-4 expression is reduced (10% remaining expression), elastic fibers display occasional disruptions, most likely because fibulin-5 cannot compensate for the partial loss of fibulin-4. *Ltbp4S*^−/−^;*Fibulin-4*^R/R^ mice: if only Ltbp-4L is expressed and fibulin-4 expression is reduced, elastogenesis is severely impaired. Elastic fibers are severely fragmented and no intact fibers are observed. *Ltbp4S*^−/−^;*Fbln5*^−/−^ mice: if only Ltbp-4L is expressed and fibulin-5 expression is missing, an alternative elastogenesis pathway most likely involving direct interaction of Ltbp-4L and fibulin-4 leads to the formation of fibrillar elastic fibers (Dabovic et al., 2015). *Ltbp4*^−/−^ mice: if Ltbp-4L and Ltbp-4S are missing, only amorphous, non-fibrillar globular elastin structures instead of fibrillar elastic fibers are present (Bultmann-Mellin et al., 2015). Modified from Bultmann-Mellin et al. (2015), Dabovic et al. (2015) and Noda et al. (2013).

Normal elastic fiber assembly involves microaggregation of tropoelastin, linked to fibulin-4 and fibulin-5, as well as binding of this complex to Ltbp-4 and its linear deposition onto fibrillin microfibrils. Subsequent coalescence of tropoelastin takes place on these microfibrils, resulting in fibrillar elastic fibers (Fig. 3) (Bultmann-Mellin et al., 2015; Dabovic et al., 2015; Noda et al., 2013; Yanagisawa and Davis, 2010). *Ltbp4S*^−/−^ mice showed short intact elastic fibers dispersed between amorphous, non-fibrillar elastin aggregates. In contrast, *Ltbp4S*^−/−^;*Fbln5*^−/−^ mice, which express Ltbp-4L and lack fibulin-5 expression, show fibrillary intact elastic fibers. This indicates that, in *Ltbp4S*^−/−^;*Fbln5*^−/−^ mice, elastogenesis takes place through an alternative pathway most likely involving a direct interaction of Ltbp-4L and fibulin-4 (Fig. 3) (Bultmann-Mellin et al., 2015; Dabovic et al., 2015). Both Ltbp-4L and Ltbp-4S interact with fibulin-4 and fibulin-5. However, Ltbp-4L has a higher affinity for fibulin-4 and fibulin-5 than does Ltbp-4S (Bultmann-Mellin et al., 2015). Interestingly, fibulin-4 deposition seems to be fibrillar, whereas fibulin-5 deposition is non-fibrillar and patchy in *Ltbp4S*^−/−^ mice, indicating that the presence of Ltbp-4L is required for proper fibulin-4 deposition (Bultmann-Mellin et al., 2015; Noda et al., 2013). Taken together, these data suggest that amorphous, non-fibrillar elastin aggregates predominantly consist of tropoelastin and fibulin-5. If fibulin-5 is missing, as in *Ltbp4S*^−/−^;*Fbln5*^−/−^ mice, these amorphous elastin aggregates cannot form and are therefore unable to interfere with the proposed alternative elastogenesis pathway involving the Ltbp-4L–fibulin-4 axis (Dabovic et al., 2015). Therefore, it can be speculated that Ltbp-4L prefers the interaction with fibulin-4 and not with fibulin-5 *in vivo*. In contrast, Ltbp-4S might favor the interaction with fibulin-5 *in vivo* because fibulin-5 deposition is amorphous and non-fibrillar in *Ltbp4S*^−/−^ mice (Bultmann-Mellin et al., 2015; Noda et al., 2013). In aortas of *Fibulin-4*^R/R^ mice, which occasionally showed elastic fiber disruptions, we found upregulated *Ltbp4S* mRNA expression, eventually representing a compensatory mechanism to restore intact elastic lamellae. However, it is apparent that Ltbp-4S interacting with fibulin-5 cannot fully compensate for the partial loss of fibulin-4 in the assembly process of intact elastic fibers (Fig. 3). In *Ltbp4S*^−/−^;*Fibulin-4*^R/R^ mice, which only expressed Ltbp-4L and 10% fibulin-4, elastic fibers are severely fragmented and no intact fibers were observed (Fig. 3). This is very similar to the amorphous, non-fibrillar globular elastin deposits seen in *Ltbp4*^−/−^ mice. In these mice, fibulin-4 is expressed, but its matrix incorporation is defective (Bultmann-Mellin et al., 2015). Together, this implies that intact fibulin-4 matrix incorporation is essential for elastic fiber assembly through an Ltbp-4L–fibulin-4-dependent pathway. Furthermore, fibulin-5 might not be able to compensate for the lack of fibulin-4 and might be incapable of interacting with Ltbp-4L in *Ltbp4S*^−/−^;*Fibulin-4*^R/R^ mice (Fig. 3).

In summary, we show that a functional interaction between Ltbp-4L and fibulin-4 is crucial for survival and elastogenesis in *Ltbp4S*^−/−^ mice. We conclude that fibulin-5 cannot compensate for the partial loss of fibulin-4 in *Ltbp4S*^−/−^;*Fibulin-4*^R/R^ mice with respect to survival and elastic fiber assembly. However, fibulin-4 can compensate for the loss of fibulin-5 because *Ltbp4S*^−/−^;*Fbln5*^−/−^ mice show rescued elastogenesis and survival (Dabovic et al., 2015). Therefore, we suggest that Ltbp-4L is required for matrix deposition of fibulin-4, whereas Ltbp-4S might target preferentially fibulin-5.

## MATERIALS AND METHODS

### Animals, breeding and genotyping

Generation and genotyping of *Ltbp4S*^−/−^, *Fibulin-4*^R/R^ and *Ltbp4*^−/−^ mice were described earlier (Bultmann-Mellin et al., 2015; Hanada et al., 2007; Sterner-Kock et al., 2002). All primers are listed in Table S1. Animals were housed in a 12-h light-dark cycle and were fed a standard rodent diet (Altromin Spezialfutter, Lage, Germany).

Unless otherwise stated, animals were sacrificed at postnatal day 4 (P4) by decapitation and autopsied using standard protocols. Lungs and abdominal aortas were used for further analyses. All animal procedures were performed in accordance with the German Laws for Animal Protection and were approved by the Institutional Animal Care and Use Committee: Landesamt für Natur, Umwelt und Verbraucherschutz Nordrhein-Westfalen (Recklinghausen, Germany).

### mRNA expression analysis

Total RNA isolation and real-time PCR were performed as described (Bultmann et al., 2013). All primers are listed in Table S1. Relative expression of *Ltbp-4L*, *Ltbp-4S*, *Efemp2*, *Ctgf* and *Pai1* was adjusted for total RNA content by glyceraldehyde-3-phosphate dehydrogenase (*Gapdh*) expression. Calculations were performed by a comparative 2^−ΔΔC^_T_ method (Livak and Schmittgen, 2001).

### SDS-PAGE and immunoblotting

Protein expression levels were determined by western blotting, using SDS-PAGE as previously described (Bultmann et al., 2013). All primary antibodies are listed in Table S2.

### Histology and immunohistochemistry

Tissue architecture and localization of proteins were analyzed using previously described histological and immunohistochemical stainings (Bultmann-Mellin et al., 2015). All primary antibodies are listed in Table S2.

### Statistical evaluation

Data are presented as mean±s.d. Differences between groups were analyzed by log-rank test or two-way ANOVA, followed by Bonferroni correction as appropriate. Statistical significance of post-hoc analyses were defined as *P*-values of *P<0.05 and **P<0.01. Calculations were performed using SPSS22 (IBM Deutschland, Ehningen, Germany).
